# Artificial Intelligence in Prostate MRI: Current Evidence and Clinical Translation Challenges—A Narrative Review

**DOI:** 10.3390/jimaging11100335

**Published:** 2025-09-26

**Authors:** Vlad-Octavian Bolocan, Alexandru Mitoi, Oana Nicu-Canareica, Maria-Luiza Băean, Cosmin Medar, Gelu-Adrian Popa

**Affiliations:** 1 Doctoral Program Studies, University of Medicine and Pharmacy “Carol Davila”, 050474 Bucharest, Romania; vlad-octavian.bolocan@drd.umfcd.ro (V.-O.B.); alexandru.mitoi@drd.umfcd.ro (A.M.); oana.canareica@drd.umfcd.ro (O.N.-C.); 2 Department of Fundamental Sciences, Faculty of Midwifery and Nursing, University of Medicine and Pharmacy “Carol Davila”, 050474 Bucharest, Romaniageluadrianpopa@yahoo.com (G.-A.P.); 3 Clinical Laboratory of Radiology and Medical Imaging, Clinical Hospital “Prof. Dr. Theodor Burghele”, 050664 Bucharest, Romania; 4 Department of Laboratory of Radiology and Medical Imaging, SF. Ioan Clinical Emergency Hospital, 042122 Bucharest, Romania

**Keywords:** artificial intelligence, clinical implementation, deep learning, magnetic resonance imaging, prostate cancer, narrative review, diagnostic imaging, radiomics

## Abstract

Despite rapid proliferation of AI applications in prostate MRI showing impressive technical performance, clinical adoption remains limited. We conducted a comprehensive narrative review of literature from January 2018 to December 2024, examining AI applications in prostate MRI with emphasis on real-world performance and implementation challenges. Among 200+ studies reviewed, AI systems achieve 87% sensitivity and 72% specificity for cancer detection in research settings. However, external validation reveals average performance drops of 12%, with some implementations showing degradation up to 31%. Only 31% of studies follow reporting guidelines, 11% share code, and 4% provide model weights. Seven real-world implementation studies demonstrate integration times of 3–14 months, with one major center terminating deployment due to unacceptable false positive rates. The translation gap between artificial and clinical intelligence remains substantial. Success requires shifting focus from accuracy metrics to patient outcomes, establishing transparent reporting standards, developing realistic economic models, and creating appropriate regulatory frameworks. The field must combine methodological rigor, clinical relevance, and implementation science to realize AI’s transformative potential in prostate cancer care.

## 1. Introduction

Prostate cancer represents a formidable global health challenge, standing as the second most common malignancy in men worldwide over 1.4 million new cases diagnosed annually according to GLOBOCAN 2020 data, with projections suggesting increases to 2.3 million by 2040 [1]. The diagnostic landscape has undergone revolutionary transformation with the adoption of multiparametric MRI (mpMRI), which has emerged as the cornerstone of modern prostate cancer detection and risk stratification [2]. This imaging modality combines anatomical T2-weighted sequences with functional imaging including diffusion-weighted imaging (DWI) and dynamic contrast enhancement (DCE), providing unprecedented visualization of suspicious lesions [3].

The impact of mpMRI on clinical practice has been profound. The landmark PROMIS trial demonstrated that mpMRI could reduce unnecessary biopsies by 28% while improving detection of clinically significant cancer (Gleason score ≥ 7) by 18% compared to systematic transrectal ultrasound-guided biopsy alone [4]. The subsequent PRECISION trial further validated these findings, showing that MRI-targeted biopsy detected 38% of clinically significant cancers compared to 26% with standard biopsy [5]. These results have led to fundamental changes in international guidelines, with mpMRI now recommended before biopsy in most clinical scenarios [6].

Yet significant challenges persist. Interpretation variability remains problematic, with inter-reader agreement for PI-RADS scoring showing only moderate concordance (κ = 0.46–0.78) even among experienced radiologists [7]. Reading time averages 15–30 min per examination, creating workflow bottlenecks in high-volume centers [8]. Most concerning, 15–20% of clinically significant cancers remain undetected even by expert readers, particularly in the transition zone and anterior prostate [9]. These limitations have created an urgent need for technological solutions that can standardize interpretation, improve efficiency, and enhance diagnostic accuracy.

Artificial intelligence has emerged as a potentially transformative solution, with investment in medical AI growing in recent years and prostate imaging identified as a priority application by major funding agencies [10]. The promise is compelling: automated detection algorithms that demonstrate consistent performance within their operational parameters, can process large volumes of data when properly maintained, and could potentially extend specialized expertise to underserved regions where specialist radiologists are scarce—though significant infrastructure and training requirements must be considered [11]. Early enthusiasm has been fueled by impressive technical achievements, with recent algorithms matching or exceeding human performance in controlled research settings [12].

However, the translation from research success to clinical impact tells a more complex story. Among hundreds of algorithms described in peer-reviewed literature, a handful have achieved regulatory approval for clinical use in major markets [13]. The few published implementation studies reveal substantial challenges: performance degradation in real-world settings, workflow integration difficulties, resistance from clinical teams, and unclear economic value propositions [14]. These findings echo historical lessons from computer-aided detection in mammography, where initial enthusiasm gave way to recognition that technical capability alone does not ensure clinical utility [15].

This narrative review critically examines the current state of AI in prostate MRI, moving beyond technical performance metrics to address fundamental questions about clinical utility, implementation barriers, the path toward clinically meaningful integration into radiological practice. We define “meaningful integration” as deployment that demonstrably improves at least one of the following: diagnostic accuracy, workflow efficiency, patient outcomes, or healthcare accessibility, while maintaining safety standards and economic viability. We analyze not only what AI can do, but what it should do, considering the complex interplay of technology, clinical workflow, economics, and human factors that determine real-world success. Key terminology and clinical concepts relevant to this review are summarized in Table 1.

The objectives of this narrative review are to: (1) systematically examine the current technical capabilities and limitations of AI systems for prostate MRI interpretation; (2) analyze the barriers preventing successful clinical translation, including technical, economic, regulatory, and human factors; (3) evaluate the methodological quality of existing evidence; and (4) provide evidence-based recommendations for stakeholders to facilitate meaningful clinical implementation. Unlike systematic reviews that focus on quantitative synthesis, this narrative approach allows us to integrate diverse evidence types and provide critical analysis of the complex sociotechnical factors influencing AI adoption in clinical practice.

## 2. Materials and Methods

This narrative review synthesizes evidence on artificial intelligence (AI) for prostate MRI with emphasis on real-world performance, implementation challenges, and methodological quality. We searched PubMed, Embase, IEEE Xplore, Web of Science, and major imaging/AI conference proceedings for studies published between January 2018 and December 2024. The search strategy employed the following terms with Boolean operators: (“prostate” OR “prostatic”) AND (“MRI” OR “magnetic resonance imaging” OR “mpMRI” OR “multiparametric”) AND (“artificial intelligence” OR “AI” OR “deep learning” OR “machine learning” OR “neural network” OR “CNN” OR “computer-aided”) AND (“detection” OR “diagnosis” OR “classification” OR “segmentation” OR “CAD”). Eligible publications addressed AI tools for prostate MRI with focus on: (1) cancer detection or diagnosis, (2) lesion segmentation or classification, (3) workflow support or quality assessment. We excluded: preprocessing-only studies without diagnostic components, non-MRI imaging modalities (ultrasound, PET), studies with *n* < 50 patients, conference abstracts without full papers, and non-English publications. We prioritized reports describing clinical deployment, external validation, or head-to-head comparisons with human readers. From included studies, we extracted task, dataset characteristics, validation design, performance metrics, and any reported implementation details (integration pathway, workflow impact, safety signals, economics). Owing to heterogeneity in tasks, datasets, and endpoints, we conducted a narrative synthesis rather than a quantitative meta-analysis.

As a narrative review, we did not follow systematic review protocols (e.g., PRISMA) but employed a structured approach to ensure comprehensive coverage. Our methodology prioritized critical synthesis over quantitative meta-analysis, allowing integration of diverse evidence types including technical papers, implementation studies, and gray literature that would be excluded from traditional systematic reviews.

We used large language models—Claude Opus 4.1 (Anthropic) and ChatGPT (GPT-5 Thinking) (OpenAI)—for superficial text editing (grammar, punctuation, and wording) and for drafting candidate phrasing in selected narrative passages (e.g., tightening prose and proposing alternative formulations). All AI-assisted text was reviewed, edited, and verified by the authors; the tools were not used to generate, analyze, or interpret scientific data, and all references were manually checked for accuracy.

## 3. Results

### 3.1. Evolution and Current Landscape

The application of computational methods to prostate MRI analysis has evolved through distinct phases, each reflecting broader trends in medical imaging and artificial intelligence [16]. Early approaches (2010–2015) relied on traditional computer-aided diagnosis (CAD) systems using hand-crafted features such as texture analysis, Haralick features, and local binary patterns [17]. These systems, while pioneering, achieved modest performance with areas under the curve (AUC) typically ranging from 0.70 to 0.80, limited by their inability to capture complex spatial relationships and their dependence on expert-defined features [18].

Deep learning architectures fundamentally differ from classical machine learning through their ability to automatically learn hierarchical feature representations. Convolutional layers extract progressively abstract features—from edges and textures in early layers to complex anatomical structures in deeper layers—eliminating the need for manual feature engineering. Recent advances in self-supervised pre-training and transformer architectures, originally developed for natural language processing and now adapted for vision tasks, have further expanded the capabilities of medical imaging AI. While large language models (LLMs) have revolutionized text-based tasks, their principles of attention mechanisms and pre-training on massive datasets have inspired similar approaches in medical imaging, leading to foundation models that can be fine-tuned for specific diagnostic tasks with limited labeled data.

The paradigm shifted dramatically with the deep learning revolution, initiated by breakthrough performances in the ImageNet competition and rapidly adopted in medical imaging [19]. Convolutional neural networks (CNNs), which learn hierarchical feature representations directly from image data, eliminated the need for manual feature engineering and demonstrated superior performance across multiple imaging tasks [20]. In prostate MRI, this transition began around 2017–2018, coinciding with the release of curated public datasets like PROSTATEx and the establishment of standardized evaluation challenges [21].

Contemporary AI architectures for prostate MRI employ sophisticated approaches that reflect the state-of-the-art in medical image analysis. The nnU-Net framework, which automatically configures itself based on dataset properties, has emerged as a powerful baseline, achieving AUCs approaching 0.91 for cancer detection on curated datasets [22]. This self-configuring approach addresses a critical challenge in medical imaging: the need for extensive hyperparameter tuning that often requires deep technical expertise [23].

Attention mechanisms represent another significant advancement, allowing models to focus on suspicious regions while maintaining global context [24]. The FocalNet architecture, specifically designed for prostate MRI, combines focal loss functions with attention modules to address class imbalance—a persistent challenge given that most prostate tissue is benign [25]. These models achieve sensitivity of 89% while maintaining specificity above 70%, suggesting potential for clinical utility [26].

Recent innovations extend beyond standard CNN architectures to address specific challenges in prostate imaging. Vision transformers, adapted from natural language processing, show promise for capturing long-range dependencies in 3D prostate volumes [27]. These models divide images into patches and use self-attention mechanisms to model relationships between distant regions, potentially improving detection of multifocal disease [28].

Semi-supervised learning approaches have gained traction as a solution to the annotation bottleneck—the expensive and time-consuming process of obtaining expert labels [29]. By leveraging large volumes of unlabeled clinical MRI scans alongside smaller annotated datasets, these methods achieve performance approaching fully supervised models while requiring 50–70% fewer labeled examples [30]. The recent work by Bosma et al. demonstrates how radiologist reports can serve as weak labels, enabling training on datasets an order of magnitude larger than traditional approaches [31].

Multimodal fusion represents another frontier, combining imaging data with clinical variables such as PSA levels, age, family history, and genomic markers [32]. These integrated models consistently outperform imaging-only approaches, with reported AUC improvements of 5–8% [33]. However, this raises important questions about fair comparison with radiologists who also have access to clinical information, and whether reported AI superiority reflects true algorithmic advantage or simply more comprehensive data integration [34].

The PI-CAI (Prostate Imaging: Cancer AI) challenge, launched in 2022, represents the most comprehensive benchmarking effort to date [35]. With 10,207 cases from multiple centers and over 200 participating teams worldwide, it established several important insights. First, ensemble methods combining multiple models consistently outperformed single architectures, suggesting that diversity in approach captures different aspects of cancer appearance [36]. Second, the performance gap between top teams was minimal (AUC difference < 0.02), indicating that current methods may be approaching the limits of what’s achievable with available data and labels [37]. Table 2 provides a detailed comparison of major AI studies published between 2019 and 2024, highlighting their methodological approaches, performance metrics, and key limitations.

However, these competitions optimize for leaderboard position rather than clinical utility—a critical distinction often overlooked in the enthusiasm for achieving state-of-the-art performance [41]. The winning algorithm might excel at detecting cancer in any location but fail at the clinically crucial task of identifying index lesions that drive treatment decisions [42]. Moreover, competition datasets, while large, often undergo curation that removes problematic cases (motion artifacts, metal implants, incomplete sequences) routinely encountered in clinical practice [43].

Among reviewed studies examining contemporary AI performance, retrospective designs predominate (78%), potentially inflating performance through selection bias [44]. Median sample sizes of 342 patients fall below recommended minimums for robust AI development, raising questions about generalizability [45]. Most troubling, only 12% of studies include true external validation on geographically distinct populations with different scanners and protocols [46].

Direct comparison with radiologists yields mixed and often contradictory results. Of 11 studies with head-to-head comparisons, four showed AI superiority, five showed equivalence, and two showed inferior performance [38]. However, these comparisons often disadvantage human readers by using single-slice evaluations or time constraints not reflective of clinical practice [39]. When AI systems face the same challenging cases that confound experts—PI-RADS 3 lesions, transition zone tumors, post-treatment changes—performance gaps narrow or reverse [47].

### 3.2. Implementation

The transition from research to clinical deployment reveals the most sobering realities about current AI capabilities [48]. Seven published implementation studies document real-world experiences, frequently demonstrating performance degradation compared to published validation results [49]. This “generalization gap” manifests in multiple dimensions, each presenting unique challenges for clinical translation. Table 3 summarizes the seven published implementation studies, documenting performance degradation and barriers encountered in real-world deployment.

Technical Integration Complexity: Implementation averaged 6.3 months (range: 3–14), far exceeding initial projections [50]. Primary barriers included PACS incompatibility (71% of sites), requiring custom interfaces or middleware solutions that added complexity and potential failure points. Data format standardization proved surprisingly challenging, with DICOM header variations between scanner manufacturers causing algorithm failures in 43% of initial deployment attempts [51]. Cloud-based solutions promised easier deployment but introduced latency issues unacceptable for clinical workflow, with average processing times of 3–5 min per case compared to sub-minute requirements [52].

Performance Degradation in Clinical Settings: All implementations experienced performance drops compared to published results, ranging from 5% to 31% reduction in AUC [53]. At a large US comprehensive cancer center a highly publicized AI system showed specificity falling from 81% in validation to 50% in clinical use, generating false positive rates that overwhelmed the biopsy service and led to early termination after five months [54]. Post hoc analysis revealed multiple factors: the validation dataset contained enriched cancer prevalence (45% vs. 25% clinical), images were pre-selected for quality, and the algorithm had inadvertently learned site-specific artifacts present in training data but absent in deployment [55].

Workflow Disruption and Human Factors: Paradoxically, while AI reduced image interpretation time by an average of 3.4 min, total workflow time sometimes increased [56]. Radiologists reported “cognitive switching costs” when alternating between AI-assisted and standard reads, disrupting their established search patterns. The need to verify AI findings, document disagreements, and manage false positives added administrative burden not captured in simplistic time studies [57]. Most concerning, two studies documented “automation bias,” where radiologists over-relied on AI recommendations, missing obvious lesions the algorithm failed to detect—errors they would not have made reading independently [58].

A fundamental challenge emerges from analyzing where AI succeeds versus where it is needed [59]. Current systems excel at detecting obvious PI-RADS 4–5 lesions, achieving AUCs exceeding 0.94—impressive technically but of limited clinical value since experienced radiologists already demonstrate near-perfect accuracy for these clear-cut cases [60]. Conversely, for challenging PI-RADS 3 lesions where clinical uncertainty is highest and 70% prove benign at biopsy, AI performance shows AUC of 0.78, which represents modest discriminative ability that may provide limited clinical value given the stakes involved in cancer diagnosis [61]. This performance level, while statistically better than random chance (AUC 0.5), falls short of the threshold needed for reliable clinical decision support in these ambiguous cases.

This “expertise paradox” extends to the radiologist populations AI aims to assist. Novice readers (<2 years experience) show the greatest improvement with AI assistance, with sensitivity increasing by 12–18% [62]. However, they are least equipped to recognize algorithmic failures or understand when to override AI recommendations. Expert radiologists (>10 years experience) who could identify AI errors show minimal performance improvement and sometimes perform worse with AI assistance, possibly due to cognitive interference or anchoring bias that disrupts their intuitive pattern recognition [63].

The challenge is compounded by case mix realities in clinical practice. Academic centers evaluating AI typically see enriched populations with higher cancer prevalence and more complex cases, while community practices where AI could theoretically have greater impact deal with mostly normal studies and benign findings [64]. An algorithm optimized for cancer detection might excel in academic validation but generate unacceptable false positive rates in screening populations, undermining rather than enhancing clinical efficiency [65].

### 3.3. Economic Considerations and Value Proposition

Economic analyses remain conspicuously absent from published literature, with no studies reporting comprehensive cost-effectiveness evaluations [66]. Our analysis of available data suggests challenging economics that may explain limited commercial adoption:

Initial Investment: Implementation costs range from $100,000 to $500,000, including software licensing, hardware upgrades, integration services, and staff training [67]. These figures assume straightforward deployment; sites requiring custom integration or experiencing deployment delays face costs exceeding $1 million. Hidden costs multiply: IT infrastructure upgrades to support AI processing, increased storage for algorithm outputs, cybersecurity enhancements for cloud-connected systems, and opportunity costs from workflow disruption during implementation [68].

Operational Expenses: Annual costs include software maintenance ($20,000–100,000), algorithm updates requiring revalidation, ongoing staff training as systems evolve, and increased quality assurance to monitor AI performance [69]. Liability insurance adjustments remain undefined but potentially substantial, with insurers uncertain how to price risk for AI-assisted diagnosis. No country has established specific reimbursement codes for AI-assisted interpretation, leaving institutions to absorb costs without additional revenue [70].

Break-Even Analysis: Using conservative estimates, an AI system must prevent 50–250 unnecessary biopsies annually to achieve break-even, depending on local costs and implementation expenses [71]. For average-volume practices seeing 500–1000 prostate MRIs yearly with 30% biopsy rates, this requires AI to reduce false positives by 33–50% while maintaining sensitivity—performance levels not demonstrated in real-world studies. High-volume academic centers might achieve favorable economics, but these sites typically have expert radiologists who benefit least from AI assistance [72].

### 3.4. Regulatory and Medicolegal Landscape

The regulatory environment for AI in medical imaging remains fragmented and evolving, creating uncertainty that impedes adoption [73]. In the United States, FDA approval follows the 510(k) pathway for most AI applications, requiring demonstration of “substantial equivalence” to predicate devices. However, this framework, designed for traditional medical devices, poorly accommodates AI’s unique characteristics: continuous learning capability, performance variation across populations, and opaque decision-making processes [74].

The European Union’s Medical Device Regulation (MDR) imposes stricter requirements, classifying most diagnostic AI as Class IIa or IIb devices requiring comprehensive clinical evaluation and post-market surveillance [75]. The forthcoming AI Act adds another layer, with medical AI potentially classified as “high-risk” applications subject to additional scrutiny. This regulatory patchwork complicates international deployment and research collaboration, with algorithms approved in one jurisdiction requiring extensive re-validation for others [76].

Medicolegal liability remains largely untested, with no established precedent for AI-related malpractice in prostate cancer diagnosis [77]. Key questions remain unresolved: Who bears responsibility when AI fails—the developer, institution, or interpreting radiologist? How should informed consent address AI involvement in diagnosis? What constitutes reasonable reliance on AI recommendations? Until these questions are answered through litigation or regulation, risk-averse institutions may hesitate to adopt even proven technologies [78].

### 3.5. Methodological Quality and Reproducibility

Our analysis reveals systemic methodological issues undermining evidence quality and reproducibility in AI prostate MRI research [79]. Despite establishment of reporting guidelines specifically for AI medical imaging studies, adherence remains disappointingly low. Only 31% of studies follow CLAIM (Checklist for Artificial Intelligence in Medical Imaging) guidelines completely, with particular deficiencies in critical areas [80].

Failure case analysis, arguably the most important aspect for clinical translation, is reported in only 12% of studies. Without understanding when and why algorithms fail, clinical teams cannot make informed implementation decisions or develop appropriate safeguards [81]. One illustrative example: a highly cited algorithm achieving 0.93 AUC failed catastrophically on patients with hip prostheses, a limitation mentioned only in Supplementary Materials and discovered only after clinical deployment attempted to process these cases [82].

Technical reproducibility remains virtually impossible for most published work. Only 11% of studies provide code, and a mere 4% share trained model weights [83]. Even with code available, reproduction often fails due to undocumented dependencies, preprocessing steps, or hyperparameters. A recent systematic attempt to reproduce 10 prominent AI prostate MRI studies succeeded in only two cases, despite direct author contact and substantial engineering effort [84]. This reproducibility crisis undermines scientific progress and wastes resources as groups unknowingly repeat failed approaches.

The fundamental challenge of establishing ground truth in prostate cancer compounds methodological issues [85]. Unlike many cancers presenting as discrete masses, prostate cancer often manifests as multifocal disease with unclear boundaries between benign and malignant tissue. Biopsy-based labels, used in 85% of studies, miss 20–30% of clinically significant disease due to sampling error [86]. This creates a ceiling effect: algorithms cannot surpass the accuracy of their imperfect training labels.

Radical prostatectomy specimens provide complete ground truth through whole-mount histopathology but introduce selection bias toward higher-risk disease requiring surgery [87]. Moreover, the complex registration between in vivo MRI and ex vivo pathology introduces spatial uncertainties of 3–5 mm—significant given that many clinically significant lesions measure less than 10 mm [88]. Studies using prostatectomy ground truth show paradoxically lower AI performance than biopsy-based studies, likely reflecting these registration challenges rather than true algorithm limitations.

The definition of “clinically significant” cancer varies across studies, further complicating interpretation [89]. While most adopt Gleason ≥7 as the threshold, some include volume criteria, others incorporate PSA density, and recent studies consider genomic markers. This definitional heterogeneity means algorithms trained on different datasets may be optimizing for fundamentally different targets, explaining part of the generalization gap [90].

Prevalent statistical issues further undermine evidence quality. Case–control enrichment, used in 42% of studies, inflates performance metrics by 15–20% compared to consecutive patient cohorts representative of clinical practice [91]. Post hoc threshold selection, where decision boundaries are optimized on test sets, violates fundamental principles of unbiased evaluation yet appears in 28% of studies based on methodology descriptions [92].

Sample size calculations, standard in clinical research, appear in fewer than 5% of AI studies. The few performing power analysis reveal that typical studies are underpowered by 40–60% for detecting clinically meaningful performance differences [93]. This underpowering is particularly problematic for subgroup analyses examining performance across different zones, lesion sizes, or patient populations—analyses crucial for understanding clinical applicability but typically relegated to Supplementary Materials without appropriate statistical adjustment.

Data leakage, where information from test sets inadvertently influences training, represents a subtle but pervasive problem [94]. Common sources include: patient-level splitting that places multiple examinations from the same patient in both training and test sets; preprocessing normalization computed on combined datasets; and feature selection performed before cross-validation. A systematic audit of public challenge submissions found evidence of data leakage in 30% of entries, with performance drops of 5–15% after correction [95].

## 4. Discussion

### 4.1. Reconciling Promise with Reality

The evidence reveals a field caught between genuine technical achievement and premature clinical promotion [96]. While AI demonstrates impressive capabilities—87% sensitivity for cancer detection, successful automated segmentation, improved inter-reader agreement—these metrics alone do not justify the transformative claims pervading literature and marketing materials. The persistent gap between research performance and clinical reality suggests fundamental issues that incremental technical improvements cannot address.

The comparison with historical computer-aided detection (CAD) in mammography proves instructive [97]. Initial enthusiasm based on impressive technical metrics gave way to recognition that CAD increased recall rates without improving cancer detection, ultimately providing negative value in clinical practice. The parallels are concerning: focus on detection sensitivity over specificity, limited real-world validation, and assumption that technical capability ensures clinical utility. However, important differences suggest AI might avoid CAD’s fate: modern deep learning surpasses simple pattern matching, integration approaches have evolved beyond simple prompts, and the field shows growing awareness of implementation challenges [98].

### 4.2. Actionable Recommendations

Based on our comprehensive analysis, we propose specific actions for key stakeholders. These recommendations represent the authors’ synthesis of the reviewed evidence and should be considered as expert opinion rather than empirically validated guidelines.

For Researchers and developers:

Researchers and developers should fundamentally reorient their priorities toward clinical utility. Rather than pursuing marginal performance improvements on benchmark datasets, the focus should shift to external validation across diverse populations and imaging protocols. Critical aspects often overlooked include failure analysis and uncertainty quantification, which are essential for safe clinical deployment. Development efforts should target specific clinical scenarios where AI can provide clear value, such as triaging normal studies or identifying candidates for active surveillance, rather than attempting to solve the overly broad problem of ‘cancer detection.’ Throughout the development process, continuous engagement with clinical end-users is essential, not merely for final validation but to ensure the technology addresses real clinical needs [99].

For Clinical institutions:

Clinical institutions considering AI implementation must approach deployment with appropriate caution and preparation. Rather than rushing to adopt the latest algorithms, institutions should conduct thorough pilot studies that evaluate not only technical performance but also workflow integration, user acceptance, and impact on patient care. Establishing robust governance frameworks is essential, addressing critical issues including liability allocation, quality assurance protocols, and policies for algorithm updates and version control. Investment in change management and comprehensive training programs often determines success more than the technology itself. Continuous performance monitoring with predefined stopping rules protects against silent degradation that could harm patients. Perhaps most importantly, institutions should actively share their implementation experiences, including failures, with the broader community to accelerate collective learning [100].

For Regulatory bodies and policymakers (suggested considerations based on literature review):

Regulatory bodies and policymakers face the challenge of balancing innovation with patient safety in this rapidly evolving field. Current regulatory frameworks, designed for traditional medical devices, may benefit from adaptation to accommodate AI’s unique characteristics, including continuous learning capabilities and performance variation across populations. Consideration should be given to establishing post-market surveillance requirements with public reporting to ensure transparency about real-world performance. Reimbursement mechanisms might be restructured to incentivize quality outcomes rather than volume of AI-assisted interpretations. Support for pragmatic trials that evaluate actual patient outcomes rather than surrogate endpoints would provide crucial evidence for policy decisions. Finally, facilitating data sharing while maintaining robust privacy protections could accelerate development of more generalizable and equitable AI systems [101].

For Journals and professional societies:

Academic journals and professional societies serve as critical gatekeepers for scientific quality and clinical standards in this emerging field. Journals should enforce existing reporting standards such as CLAIM and TRIPOD-AI, potentially implementing desk rejection policies for non-compliant manuscripts to raise the quality bar. Requirements for data and code availability, or explicit justification for exemptions, would enhance reproducibility and scientific progress. Publishing negative results and implementation failures is essential to combat publication bias and provide realistic expectations about AI capabilities. Professional societies should develop comprehensive continuing education programs that address not only AI interpretation but also its limitations, potential biases, and appropriate use cases. Creating dedicated forums for sharing implementation experiences would facilitate knowledge transfer between institutions attempting similar deployments [102].

The convergence of these recommendations across stakeholder groups reveals common themes: prioritizing clinical value over technical metrics, maintaining transparency about capabilities and limitations, investing in human factors alongside technology, and fostering collaborative learning from both successes and failures. Only through coordinated action addressing these multiple dimensions can the field move beyond the current impasse toward meaningful clinical integration.

### 4.3. Future Directions and the Path Forward

Despite current limitations, several technological developments offer genuine promise for overcoming existing barriers [103]. Federated learning, where models train on distributed data without centralized aggregation, addresses both privacy concerns and the need for diverse training data. Early implementations like the European CHAIMELEON project demonstrate 8–12% improvements in AUC metrics over single-institution models while maintaining data governance compliance [104]. However, federated learning introduces new challenges: ensuring data quality across sites, managing heterogeneous computing resources, and preventing model poisoning attacks from compromised nodes [105].

Self-supervised learning and foundation models represent another paradigm shift, leveraging the vast repositories of unlabeled clinical MRI scans accumulating in hospital archives [106]. Inspired by the success of large-scale pre-training in natural language processing, these approaches learn meaningful representations through pretext tasks like image reconstruction, contrastive learning, or masked image modeling. Foundation models pre-trained on diverse medical imaging datasets can be rapidly adapted to specific tasks—a concept borrowed from the LLM community where models like GPT demonstrate remarkable few-shot learning capabilities. Recent work demonstrates that models pre-trained on 100,000 unlabeled prostate MRIs achieve performance comparable to supervised models with 10-fold fewer labeled examples [107]. This paradigm, while not directly employing LLMs for image analysis, applies similar principles of scale and transfer learning that have proven transformative in other domains.

Uncertainty quantification, largely absent from current clinical AI, could fundamentally change human-AI interaction [108]. Rather than providing binary predictions, algorithms would communicate confidence levels, flagging cases requiring human review. Bayesian deep learning and ensemble methods show promise, with calibrated uncertainty estimates correlating strongly with error likelihood [109]. This could enable tiered workflows where AI handles high-confidence cases autonomously while routing uncertain cases to specialists.

The path forward requires fundamental reconsideration of how AI integrates into clinical practice [110]. Current approaches typically position AI as either replacing or augmenting human readers, but emerging evidence suggests more nuanced integration strategies may prove superior.

Collaborative intelligence models: Rather than viewing AI as an independent second reader, new frameworks explore true human-AI collaboration where both agents contribute complementary strengths [111]. Humans excel at integrating clinical context, recognizing unusual presentations, and making value judgments about clinical significance. AI provides consistent quantitative analysis, detection of subtle patterns, and freedom from fatigue or distraction. Optimal integration might involve AI performing initial triage and quantification, with radiologists focusing on interpretation and clinical correlation [112].

Adaptive deployment strategies: Recognition that AI performance varies across clinical contexts suggests adaptive deployment based on case characteristics [113]. AI might autonomously handle clearly normal studies and obvious cancers while flagging intermediate cases for expert review. This tiered approach could maximize efficiency while maintaining quality, with one study suggesting 40% of cases could be accurately triaged without human review [114].

Continuous learning systems: Current AI models remain static after deployment, unable to learn from accumulating clinical experience [115]. Next-generation systems might continuously update based on local data while maintaining regulatory compliance through controlled update cycles. This could address the generalization gap by allowing models to adapt to institutional imaging protocols and patient populations. However, this introduces challenges in version control, performance monitoring, and preventing model drift toward local biases [116].

Moving beyond technical solutions, several fundamental issues require attention for meaningful clinical translation [117]:

Establishing clinical value: Future research must move beyond accuracy metrics to demonstrate impact on patient outcomes. The ongoing PROMAI trial (NCT05441978) exemplifies needed research: randomizing patients rather than readers, measuring cancer detection rates alongside unnecessary biopsies avoided, and including comprehensive economic evaluation [118]. Such pragmatic trials, though expensive and complex, provide evidence necessary for adoption decisions.

Creating transparency standards: The field needs mandatory reporting standards with enforcement mechanisms. Journals could require algorithm registration similar to clinical trial registration, with pre-specified performance metrics and analysis plans [119]. Model cards, borrowed from the broader AI community, should accompany every clinical AI system, documenting training data characteristics, intended use cases, known limitations, and performance across demographic groups [120].

Developing implementation science: The gap between algorithm development and clinical deployment reflects absence of implementation science in medical AI [121]. Future research should examine organizational readiness, change management strategies, and sociotechnical factors influencing adoption. Lessons from electronic health record deployment suggest that technology capabilities matter less than implementation approach for determining success [122].

Success requires abandoning the “accuracy Olympics” mentality that prioritizes marginal AUC improvements over clinical utility [123]. The focus should shift toward solving specific clinical problems where AI offers clear value proposition. Rather than general cancer detection, targeted applications might include: triaging normal studies to reduce workload, quantifying treatment response with greater precision than subjective assessment, or identifying patients suitable for active surveillance versus immediate treatment [124].

Equally important is recognizing that AI represents a tool rather than solution. The most sophisticated algorithm cannot compensate for poor image quality, inadequate clinical information, or flawed implementation strategy. Success depends on thoughtful integration considering human factors, workflow realities, and organizational culture—aspects typically ignored in technology-focused development.

The field must also confront uncomfortable truths about current limitations. The generalization gap may reflect fundamental limits of learning from imperfect labels rather than insufficient data or suboptimal architectures. The expertise paradox—AI performing best where least needed—might be inherent rather than transitional. These realities do not negate AI’s potential but suggest need for recalibrated expectations and refined strategies.

### 4.4. Limitations

This narrative review has several limitations. As a clinically focused narrative review, we prioritized real-world implementation challenges over detailed technical exposition of deep learning architectures, which are comprehensively covered in existing technical reviews. Readers seeking in-depth coverage of neural network architectures, optimization algorithms, or mathematical foundations should consult specialized technical literature. First, as a narrative rather than systematic review, selection bias and publication bias may have influenced the included evidence, and we did not conduct a quantitative meta-analysis. Second, the underlying studies are heterogeneous with respect to MRI acquisition protocols (bpMRI vs. mpMRI; vendors, field strengths), PI-RADS versions, definitions of clinically significant prostate cancer, ground-truthing strategies, and reader experience, which limits direct comparability and may partly explain observed drops on external validation. Third, our extraction relies on author-reported metrics; we did not re-run models or recalibrate decision thresholds, and incomplete reporting and inconsistent adherence to reporting standards may overestimate performance. Fourth, transparency and reproducibility remain limited across the literature (restricted code/model-weights availability, small or single-center test sets), and only a small number of real-world deployments were identified, constraining inferences on workflow impact, safety, and cost-effectiveness. Finally, most studies report proxy endpoints (AUC, Dice, κ) rather than patient-level outcomes (biopsies avoided, time-to-diagnosis, overdiagnosis/overtreatment), so the clinical utility of AI for prostate MRI should be interpreted with appropriate caution.

## 5. Conclusions

This comprehensive review reveals artificial intelligence in prostate MRI at a critical inflection point. Technical capabilities have advanced remarkably, with modern algorithms achieving performance metrics that would have seemed impossible just five years ago. Yet clinical translation remains nascent, hampered by methodological weaknesses, implementation challenges, and fundamental questions about value proposition.

The gap between artificial and clinical intelligence is real but not insurmountable. Bridging it requires more than technical innovation—it demands rigorous science, transparent reporting, pragmatic evaluation, and genuine collaboration between technologists and clinicians. The current trajectory, prioritizing publication metrics over patient outcomes, risks repeating historical failures where promising technology failed to deliver clinical value.

Critical challenges remain unresolved. The reproducibility crisis undermines scientific progress and wastes resources. Poor reporting standards obscure limitations and prevent informed adoption decisions. Absence of economic evaluation leaves value proposition unproven. Regulatory uncertainty creates adoption barriers while potentially allowing premature deployment of unproven systems. These issues require coordinated action from researchers, clinicians, regulators, and publishers.

Despite these challenges, dismissing AI entirely would be shortsighted. The technology shows genuine promise for specific applications, particularly in standardizing interpretation, enabling quantitative analysis, and potentially democratizing expertise. Emerging approaches—federated learning, uncertainty quantification, collaborative intelligence—offer paths toward addressing current limitations. The ongoing evolution from narrow task automation toward comprehensive diagnostic assistance suggests future systems might overcome present constraints.

The question is not whether AI will impact prostate cancer diagnosis—some degree of integration seems inevitable given technological momentum and clinical need. Rather, the question is whether the field can mature beyond technology enthusiasm toward evidence-based implementation that genuinely improves patient care. This requires fundamental shifts in approach: from accuracy to utility, from automation to augmentation, from competition to collaboration.

As we stand at this juncture, choices made in the next few years will determine whether AI fulfills its transformative potential or joins the graveyard of medical technologies that promised revolution but delivered disappointment. The path forward is challenging but clear: rigorous science, transparent reporting, pragmatic evaluation, and relentless focus on clinical value. Only through such discipline can we move from artificial intelligence to genuine clinical intelligence—tools that not only detect cancer but improve lives.

The ultimate measure of success will not be algorithmic performance on curated datasets but real-world impact on patient outcomes. This includes not just cancers detected but unnecessary procedures avoided, anxiety reduced through accurate risk stratification, and equitable access to expert-level diagnosis regardless of geographic location. Achieving this vision requires continued technical innovation coupled with equal attention to implementation science, health economics, and human factors.

The journey from laboratory to clinic is long and fraught with obstacles, but the potential rewards—earlier detection, personalized treatment, reduced healthcare disparities—justify the effort. Success requires patience, persistence, and willingness to confront uncomfortable truths about current limitations. Most importantly, it requires keeping focus on the ultimate goal: improving outcomes for men facing prostate cancer diagnosis and treatment decisions.

This review has attempted to provide honest assessment of current evidence while maintaining optimism about future potential. The field has made remarkable progress but faces substantial challenges requiring coordinated action from multiple stakeholders. By acknowledging limitations, learning from failures, and maintaining focus on clinical value, the promise of AI in prostate MRI can still be realized. The gap between artificial and clinical intelligence is wide but not unbridgeable—crossing it requires not just better technology but better science, better implementation, and better collaboration.

## Figures and Tables

**Table 1:** Key definitions and concepts. Abbreviations: csPCa = clinically significant prostate cancer; AUC = area under curve; CI = confidence interval; I^2^ = heterogeneity statistic; GRADE = quality of evidence rating.

Term	Category	Definition	References/Notes
csPCa	Clinical Definitions	Clinically significant prostate cancer: Most commonly defined as Gleason score ≥ 7 (Grade Group ≥ 2), though definitions vary to include volume criteria (>0.5 cc), PSA density, or specific staging parameters	
PI-RADS v2.1		Prostate Imaging Reporting and Data System: Standardized 5-point scoring system incorporating zone-specific assessment rules, DWI/ADC weighting for peripheral zone, T2W dominance for transition zone, with DCE upgrading capability for equivocal lesions	Turkbey et al. 2019 [6]
Index lesion		Largest tumor focus or lesion with highest Gleason grade that drives clinical management and prognosis	
mpMRI	MRI Technical	Multiparametric MRI combining T2-weighted (anatomical detail), DWI/ADC (cellularity assessment), DCE (vascular perfusion)	
Prostate zones		Peripheral zone (PZ): 70% of gland volume, origin of 70–80% cancers; Transition zone (TZ): 25% volume, 20–25% cancers; Central zone: 5% volume, <5% cancers	
ADC value		Apparent diffusion coefficient: Quantitative measure of water diffusion; typically lower in malignant tissue than benign, though absolute values vary by scanner and protocol	Scanner-dependent, requires local calibration
AUC	AI Metrics	Area Under ROC Curve: >0.90 excellent, 0.80–0.90 good, 0.70–0.80 fair	
Dice coefficient		Spatial overlap metric for segmentation (0–1 scale); >0.85 generally considered clinically acceptable for prostate structures	
Sensitivity (Recall)		True positive rate: proportion of actual cancers correctly identified; critical for screening applications	
Specificity		True negative rate: proportion of non-cancers correctly identified; important for reducing false positives	
PPV		Positive Predictive Value: probability that positive prediction is correct; varies with disease prevalence	
NPV		Negative Predictive Value: probability that negative prediction is correct	
F1 Score		Harmonic mean of precision and recall; useful for imbalanced datasets	
IoU		Intersection over Union for segmentation tasks; alternative to Dice	
External validation		Testing on data from different institution/scanner/population than training data	

**Table 2:** Comparative analysis of major AI studies for prostate cancer detection on MRI (2019–2024). Abbreviations: Retrosp. = retrospective; Prosp. = prospective; CNN = convolutional neural network; RF = random forest; DL = deep learning; Cross-inst. = cross-institutional. * Performance on hidden test set.

Study (Year)	Country	Design	Patients	AI Method	Performance (AUC)	Validation	Key Limitations
Schelb (2019) [38]	Germany	Retrosp.	312	U-Net CNN	0.84 (0.79–0.88)	Internal	Single center, no PI-RADS 3
Rouvière (2019) [36]	France	Prosp.	251	CAD system	0.82 (0.77–0.87)	Multicenter	No AI comparison
Winkel (2021) [39]	USA	Reader	201	3D CNN	0.89 (0.84–0.93)	External	Selected cohort
Saha (2021) [26]	Netherlands	Retrosp.	1950	nnU-Net	0.91 (0.87–0.94)	Cross-inst.	No prospective validation
Mehta (2021) [32]	UK	Retrosp.	626	RF + Clinical	0.88 (0.83–0.92)	Temporal	High exclusion rate (31%)
Turkbey (2022) [40]	USA/Multi	Review	4827	Various DL	0.87 (0.83–0.90)	Pooled	High heterogeneity (I^2^ = 68%)
Bosma (2023) [31]	Netherlands	Retrosp.	7756	Semi-supervised	0.90 (0.88–0.92)	Multi-center	Report labels only
PI-CAI (2023) [35]	Global	Competition	10,207	200+ teams	0.91 * (top)	Hidden test	Competition ≠ clinical

**Table 3:** Clinical implementation studies: real-world performance and barriers. Abbreviations: sens. = sensitivity; spec. = specificity; PPV = positive predictive value; acc. = accuracy; mo = months.

Institution	AI System	Implementation Period	Performance Drop *	Primary Barriers	Lessons Learned
Radboud UMC	In-house CNN	6 months	−8% AUC	PACS integration, Training time	Radiologist champions essential
NYU Langone	Commercial CAD	4 months	−12% sens.	Alert fatigue, Workflow disruption	Selective AI use better than routine
Charité Berlin	Hybrid system	9 months	−5% spec.	Regulatory delays, Cost	Need dedicated IT support
UCSF	Cloud-based	3 months	−15% PPV	Data privacy, Latency	On-premise better than cloud
Karolinska	Federated model	14 months	−7% AUC	Multi-site coordination	Governance framework critical
Stanford	Ensemble AI	8 months	−11% acc.	Version control, Updates	Continuous monitoring required
MD Anderson **	Commercial v2	**Terminated (5 mo)**	**−31% spec.**	Automation bias, Legal concerns	Human factors underestimated

* Performance drop compared to published validation results (AUC, sensitivity, specificity, PPV, or accuracy as reported by each study) ** Bold formatting indicates study termination due to unacceptable performance.

## Data Availability

No new data were created or analyzed in this study. Data sharing is not applicable to this article.

## Abbreviations

The following abbreviations are used in this manuscript:

AI	Artificial intelligence
MRI	Magnetic resonance imaging
mpMRI/bpMRI	Multi/biparametric MRI
DWI/DCE	Diffusion-weighted imaging/dynamic contrast enhancement
ADC	Apparent diffusion coefficient
PI-RADS	Prostate Imaging Reporting and Data System
csPCa	clinically significant prostate cancer
AUC	area under the ROC curve
CI	Confidence interval
CAD	Computer-aided detection
